# Recombinase-Mediated Cassette Exchange (RMCE)-in Reporter Cell Lines as an Alternative to the Flp-in System

**DOI:** 10.1371/journal.pone.0161471

**Published:** 2016-08-19

**Authors:** Morten M. Callesen, Martin F. Berthelsen, Sira Lund, Annette C. Füchtbauer, Ernst-Martin Füchtbauer, Jannik E. Jakobsen

**Affiliations:** 1 Department of Biomedicine, Faculty of Health, Aarhus University, Aarhus C, Denmark; 2 Department of Clinical Medicine, Faculty of Health, Aarhus University, Aarhus N, Denmark; 3 Department of Molecular Biology and Genetics, Aarhus University, Aarhus C, Denmark; The Roslin Institute, UNITED KINGDOM

## Abstract

Recombinase mediated cassette exchange (RMCE) is a powerful tool for targeted insertion of transgenes. Here we describe non-proprietary 'RMCE-in' cell lines as an alternative to the 'Flp-in' system and cell lines. RMCE-in cell lines offer a number of advantages including increased efficiency of integration of the genetic element of interest (GEI) at a single docking site, lack of bacterial backbone at the docking site both before and after GEI integration, removal of selection and visual markers initially present at the docking site upon GEI integration and the possibility to validate GEI integration by loss of a red fluorescence reporter. Moreover, the RMCE-in cell lines are compatible with GEI donors used for the Flp-in system. We demonstrate a three-step procedure for generating RMCE-in cell lines, (I) RMCE-in transposon and SB10 transposase transfection, (II) clone isolation, and (III) selecting single integrated clones with highest RFP level, which could in principle be used to turn any cell line into an RMCE-in cell line. The RMCE-in system was used as a proof of concept to produce three new RMCE-in cell lines using HEK293, HeLa, and murine embryonic stem (mES) cells. The established RMCE-in cell lines and vector are freely available from the ATCC cell bank and Addgene respectively.

## Introduction

**Fig 1:**
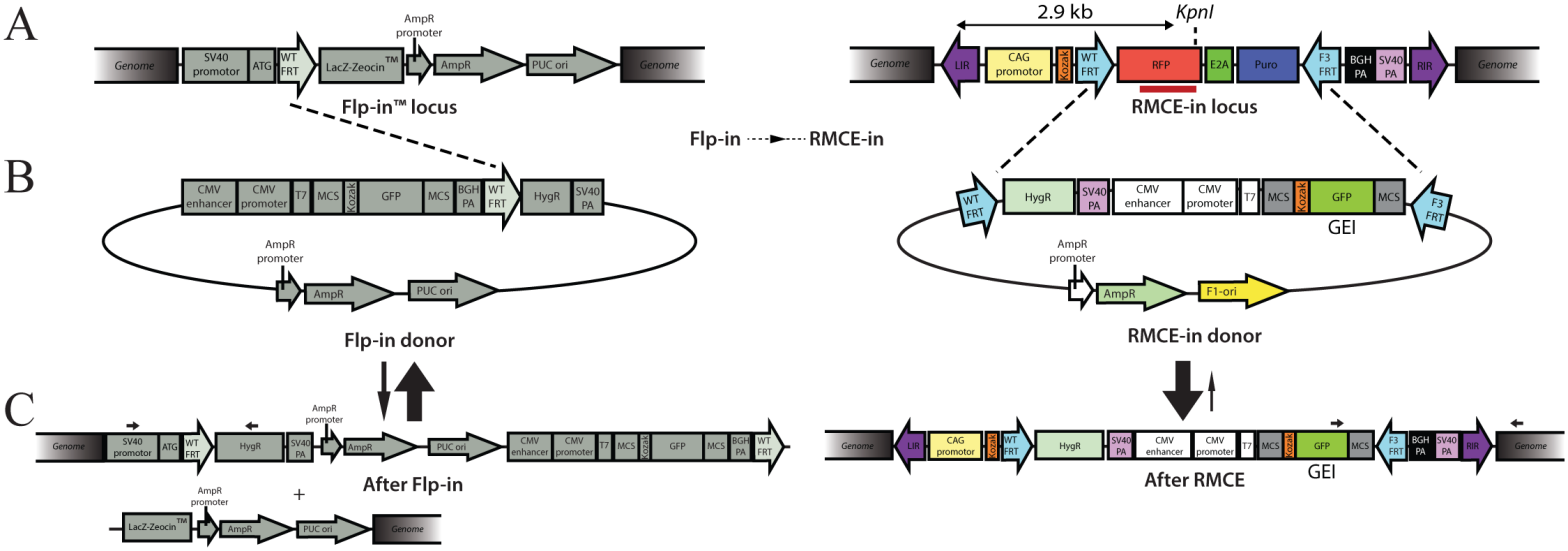
Design of the RMCE docking site. (A) *Left*: Schematics of the commercially available Flp-in docking site. *Right*: Design of the SB transposon constituting the RMCE docking site present in the new RMCE-in cell lines. The RMCE docking site contains the CAG promoter which drives the expression of a RFP reporter linked to the puromycin-resistant gene through the ribosomal skip element E2A. (B) Schematic representation of donor plasmid used for Flp-in (left) and RMCE-in (right). The genetic element of interest (GEI) is represented by a GFP reporter. Note that the GFP in the RMCE-in donor does not contain a poly(A)-signal and utilizes the poly(A) from the RMCE docking site. The RMCE-in and the Flp-in donor plasmid are compatible with both the RMCE-in and Flp-in cell line. (C) Post recombination of the Flp-in system *left*: Prokaryotic elements, the initial marker and selection gene are present in the commercial Flp-in^™^-293 cells post recombination, while the RMCE-in *right* leaves no prokaryotic DNA or initial reporter genes after cassette exchange.

Functional investigation of genetic elements such as promoters, protein coding genes, non-coding RNA, etc. in a transgenic gain-of-function approach is often hampered by the regulatory influences of sequences flanking the transgene integration site. This so-called positional effect can be circumvented by placing the genetic element of interest (GEI) in a pre-determined, fixed position in the genome. Cell lines with GEI insertion by flippase (Flp) [1] recombination in defined flippase recognition target (FRT) sites have been developed and commercialized as Flp-in^™^ cells (Fig 1 left). However, the Flp-in system succumb to a genuine problem by co-integrating several prokaryotic elements of the plasmid backbone with insertion of the GEI. In the context of GEI, such prokaryotic elements have been shown to affect the regulation of GEI expression [2–5]. In addition to the plasmid backbone, the initial lacZ-Zeocin selection marker gene also resides in the docking site with reported read-through by the Flp-in protocol [6]. Moreover, additional transgenes in the vicinity of a GEI have also been reported to affect the regulation of GEI [7]. Finally, it has been reported that the Flp-in system does not select against expression from additional, randomly integrated GEI[3,8], which may interfere and obstruct the experiments.

Recombinase-mediated cassette exchange (RMCE) offers a solution to these disadvantages of the Flp-in systems [9,10]. Based on the RMCE technology [11] we developed a vector system, that allows GEI insertion without plasmid backbone. We thereby avoid the residues of the initial selection marker and benefit from the expression being obtained from the desired insertion site. Here we present a freely available application of RMCE systems in HEK293, HeLa, and murine embryonic stem cells (mES) called 'RMCE-in'. The RMCE-in docking site was transposed with the Sleeping Beauty (SB) transposase [2,12] in HEK and HeLa cells, with little sequence dependence and a preference for open loci [13]. SB was employed rather than a, targeted approach using either viral-transduction, Zinc-finger-nucleases, TALEN or CRISPR-Cas, to eliminate the risk of off-target, but above all to produce a variety of clones with different expression levels. The RMCE-in cell lines furthermore visualize the integration of GEI by loss of a red fluorescent signal. A rather simplified system compared with Christopher et al., 2015 [14] MIN-tag system, however a functional reliable system for GEI knock-in studies. RMCE-in HEK, Hela, and mES cells are compatible with both Flp and RMCE donors. We demonstrate the difference between Flp-in and RMCE-in cell lines and describe a three-step protocol for the generation of the RMCE-in cell line of choice.

## Results

### Generation of RMCE-in cell lines

**Fig 2:**
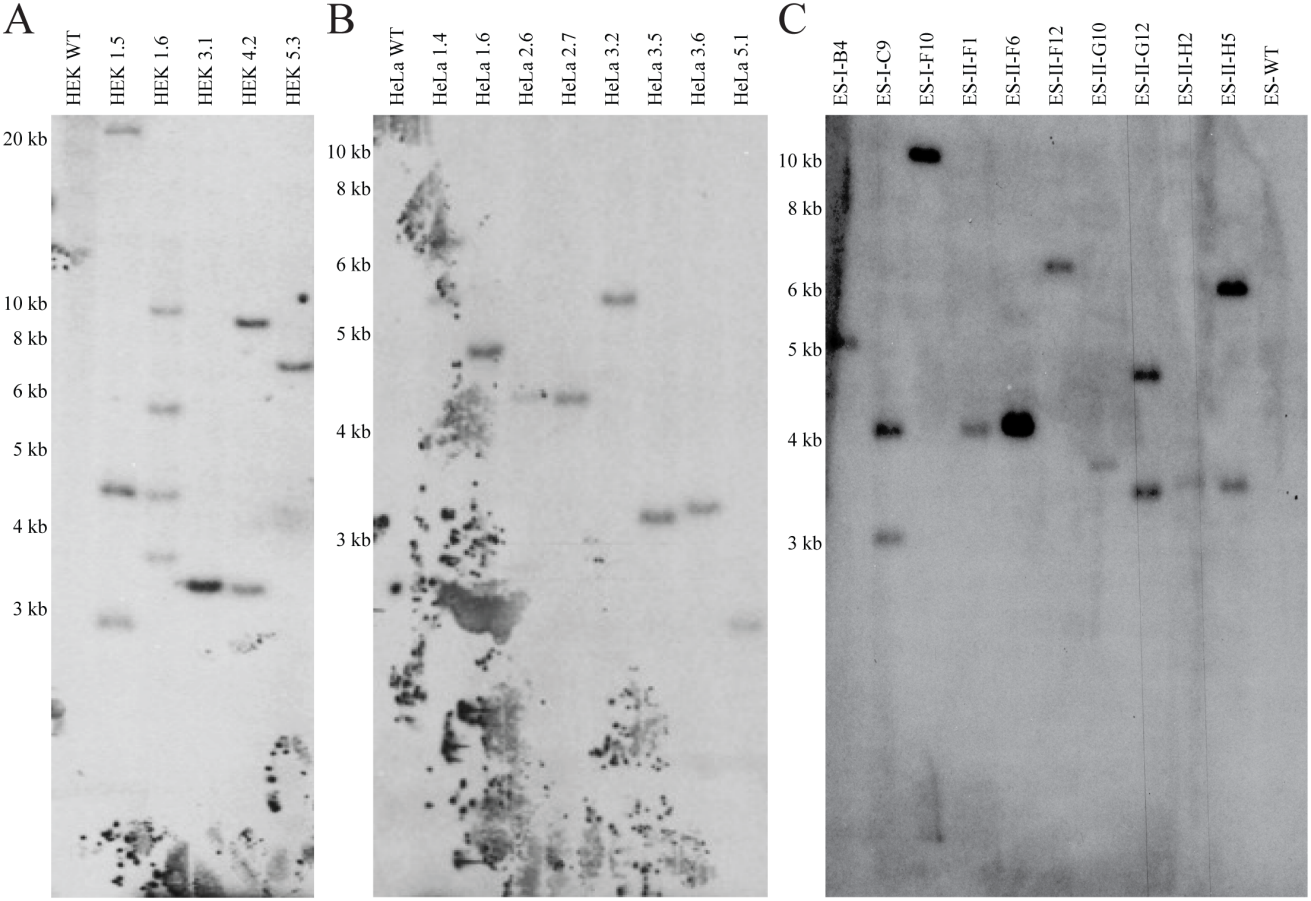
Transposon copy number in the RMCE-in HEK, HeLa, and murine ES cell clones. **(A)** Southern blot of selected genomic DNA from *KpnI* digested HEK, (**B)** HeLa and *PstI* digested (**C)** mES clones. A 700bp dCTP^32^ labeled RFP probe (red rectangle Fig 1A) identified transposition of the gene cassette at bands > 2900bp as illustrated in Fig 1A. Clone numbers are depicted above each lane.

Transposition of the RMCE-in transposon plasmid (Fig 1), was carried out by co-transfecting HEK293 cell and HeLa cells with a SB10 transposase plasmid. The mES cell clones were produced by electroporation of linearized RMCE-in transposon plasmid cleaved outside the left and right inverted repeats (LIR, RIR), to increase the likelihood of single-integrations. RMCE-in mES cells thus represents non-transposition insertion events. Twelve to thirty puromycin-resistant HEK, HeLa, and mES clones were produced. Transposon copy variations were assessed by qPCR, and compared with the reference gene GLIS3 to select clones with highest Ct values. SB integration was judged by Southern blot of DNA isolated from HEK, HeLa, and mES clones. The HEK clones carried one to four copies of the transposon (Fig 2A) while seven out of eight HeLa clones and seven out of ten mES clones were single integrated clones (Fig 2B and 2C). Note that single integrated RMCE-in transposons was established using a ratio of either 10:1 or 20:1 of the RMCE-in transposon to SB producing the HEK RMCE-in clones 3.1 and 5.3 and HeLa clones. The *KpnI* (*PstI* in mES) to LIR distance is >2900bp (Fig 1A), any bands below this size represents therefore a partial integration and falsify the clone. Southern blot assessment reveals that HeLa clone 5.1 contained a fractionated transposon and therefore not a suitable RMCE clone (Fig 2B).

**Fig 3:**
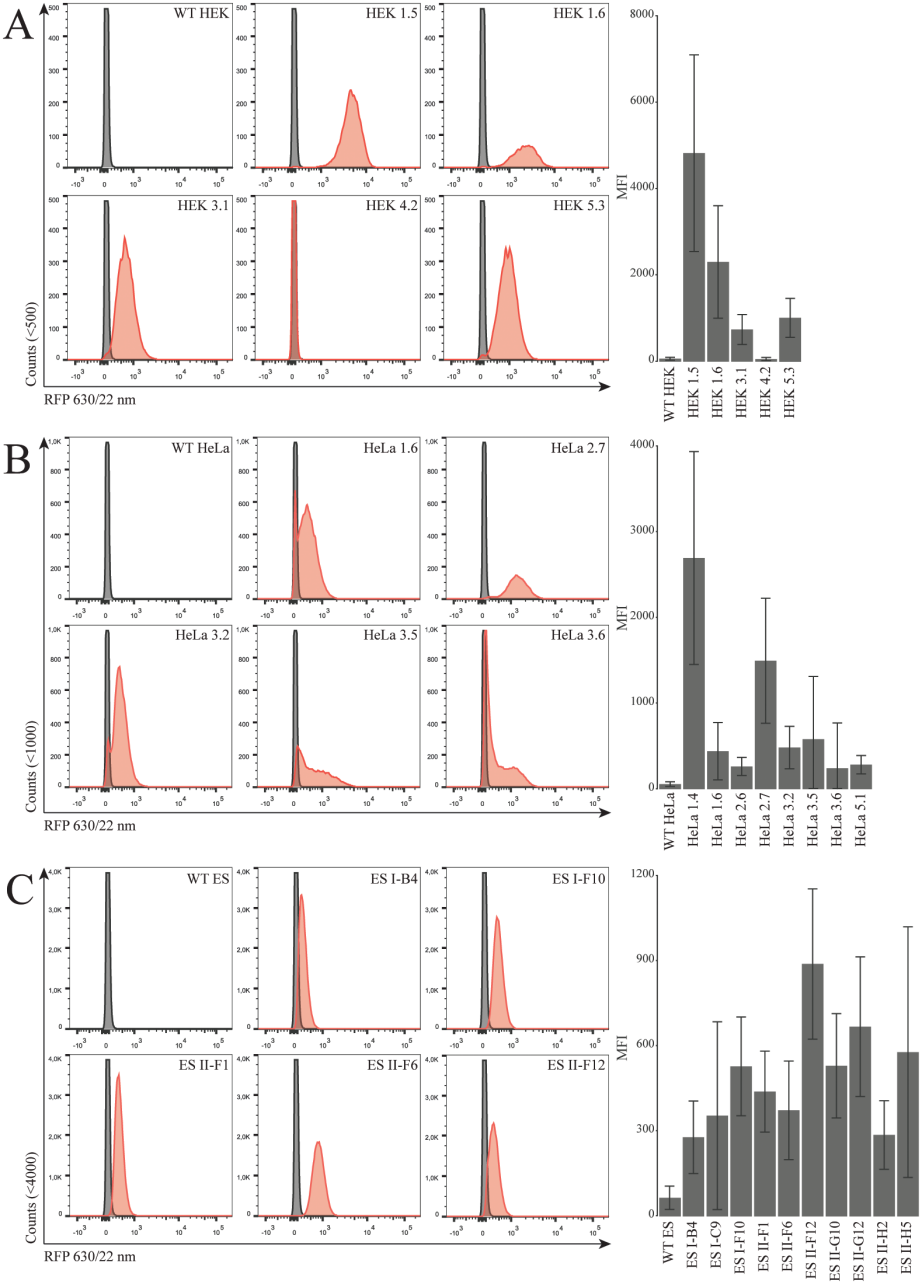
Expression from the RMCE docking site. **(A)** Representative flow cytometry results by RMCE-in from the HEK (**B)** HeLa (**C)** and mES clones. RFP emission compared with WT cells (shown in gray). All HEK, mES clones, and HeLa clone 2.7 showed a single RFP peak indicating homogenous populations. *Right*: MFI values of RMCE-in HEK, HeLa, and mES cell clones (Clone numbers correspond to the Southern blot shown in Fig 2) are shown. Cell singlet mean [min-max] counts; RMCE HEK clones 8829[3194–11875] HeLa clones 15369[5588–21004], mES clones 49757[42637–57742]

**Fig 4:**
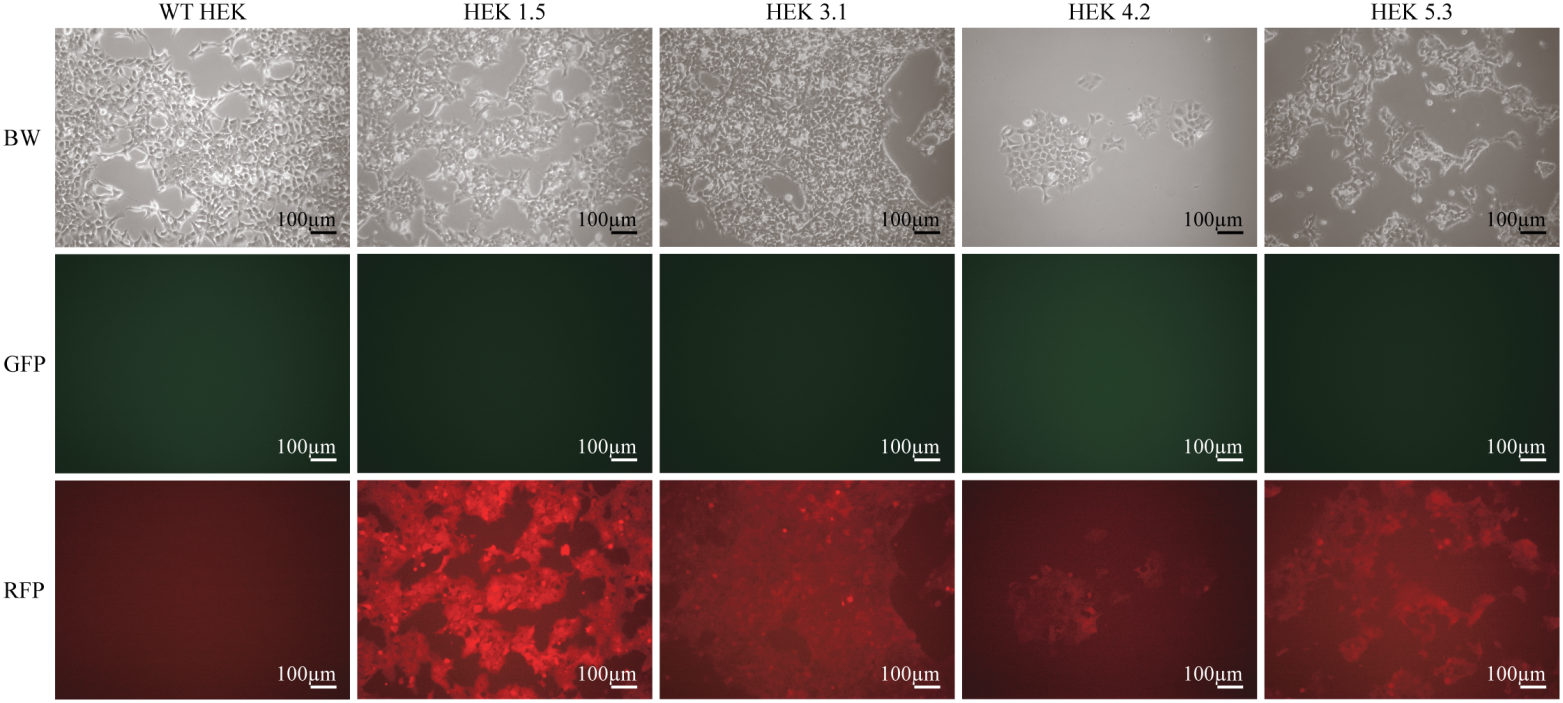
Validating flow cytometry results by fluorescence microscopy in RMCE-in HEK clones. HEK clones were exposed to 100X magnification and 1s exposure time for the assessment of RFP and GFP emission. All clones including colony 4.2 were RFP-positive and GFP with correlation to the high MFI value observed in flow cytometry (Fig 3A).

Expression level at the different insertion sites was evaluated by red fluorescence protein (RFP) signal analyzed by flow cytometry. Results from representative clones are shown in Fig 3A–3C. The majority of the HEK clones showed a uniform RFP peak indicating homogenous clones. In HEK clone 4.2, no RFP was detectable by flow cytometry (Fig 3A); however, the clone did show RFP in fluorescent microscopy (Fig 4). Many of the HeLa cell clones did not show a uniform emission pattern and contained non-fluorescent cells comparable to the WT control cells Fig 3B. This indicates that the majority of HeLa clones might not be clonal. However, HeLa clone 2.7 presented a uniform peak indicating true clonality (Fig 3B). The mES cell clones showed uniform RFP peaks indicating that all of the clones were homogenous (Fig 3C). The expression level from the integrated transposon, which constitutes the RMCE docking site, was analyzed by calculating the median fluorescent intensity (MFI) of each clone (Fig 3A–3C). The highest MFI values were observed for HEK clones 1.5 with three integrations and 1.6 with four integrations. The highest RFP MFIs from single integration were observed in HEK clone 5.3, HeLa clone 2.7, and mES clone II-F6.

**Fig 5:**
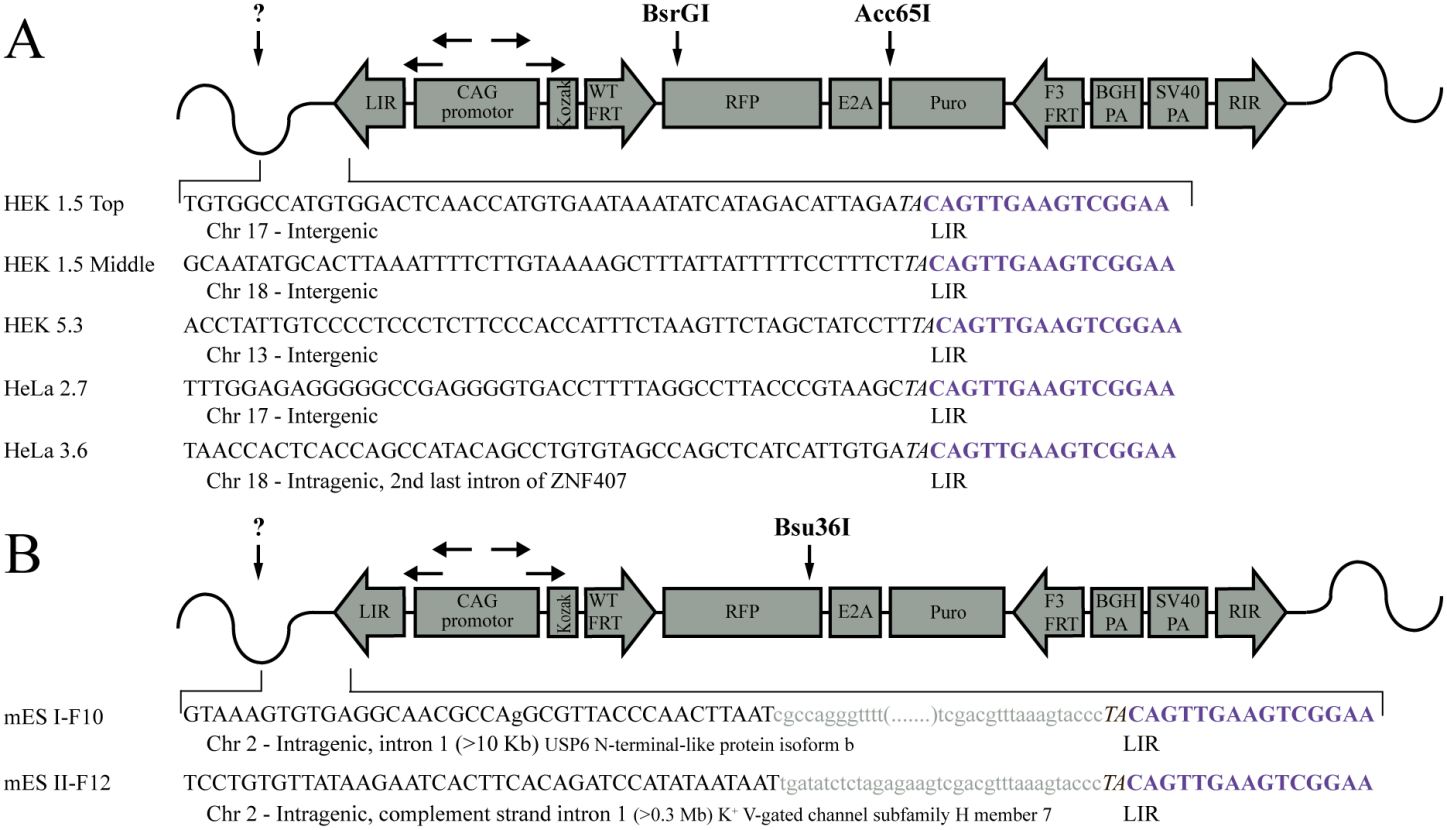
Genomic mapping of chosen clones. Long-distance inverse PCR was used to map the position of the RMCE docking site integration in HEK colony 1.5 and 5.3, HeLa colony 2.7 and 3.6 **(A)**, and mES colony II-F1, II-H2, and I-F10 **(B).** Plasmid backbone (grey) and 5’ LIR sequences (purple) obtained by Sanger sequencing are shown above the area of insertion.

The genomic localization of the RMCE docking site integration was mapped in HEK clones 1.5 and 5.3, HeLa clones 2.7 and 3.6, as well as mES cell clones I-F10 and II-F12 using long-distance inverse (LDI) PCR. The results are shown in Fig 5A and 5B. Two of the three integrations in HEK clone 1.5 were mapped to an intergenic region on chromosome 17 and 18. The single integration in HEK clone 5.3 was also found to be intergenic on chromosome 13 (Fig 5A). The single integrations found in HeLa clones 2.7 and 3.6 were mapped to an intergenic region on chromosome 17 and in the second- to last intron of the *ZNF407* gene on chromosome 18, respectively (Fig 5A). In the two mES cell clones I-F10 and II-F12, the integration sites were mapped to the second- to last intron of *USP6* N-terminal-like protein isoform b and to intron 1 of the potassium voltage-gated channel subfamily H member 7, respectively (Fig 5C). Because the HEK clone 5.3, HeLa clone 2.7, and mES clone II-F6 are apparently clonal, have a single integration, and showed the highest red fluorescent expression, they were expanded and chosen as new RMCE-in cell lines.

### Comparing the HEK293 RMCE-in with the HEK293 Flp-in cell line

**Fig 6:**
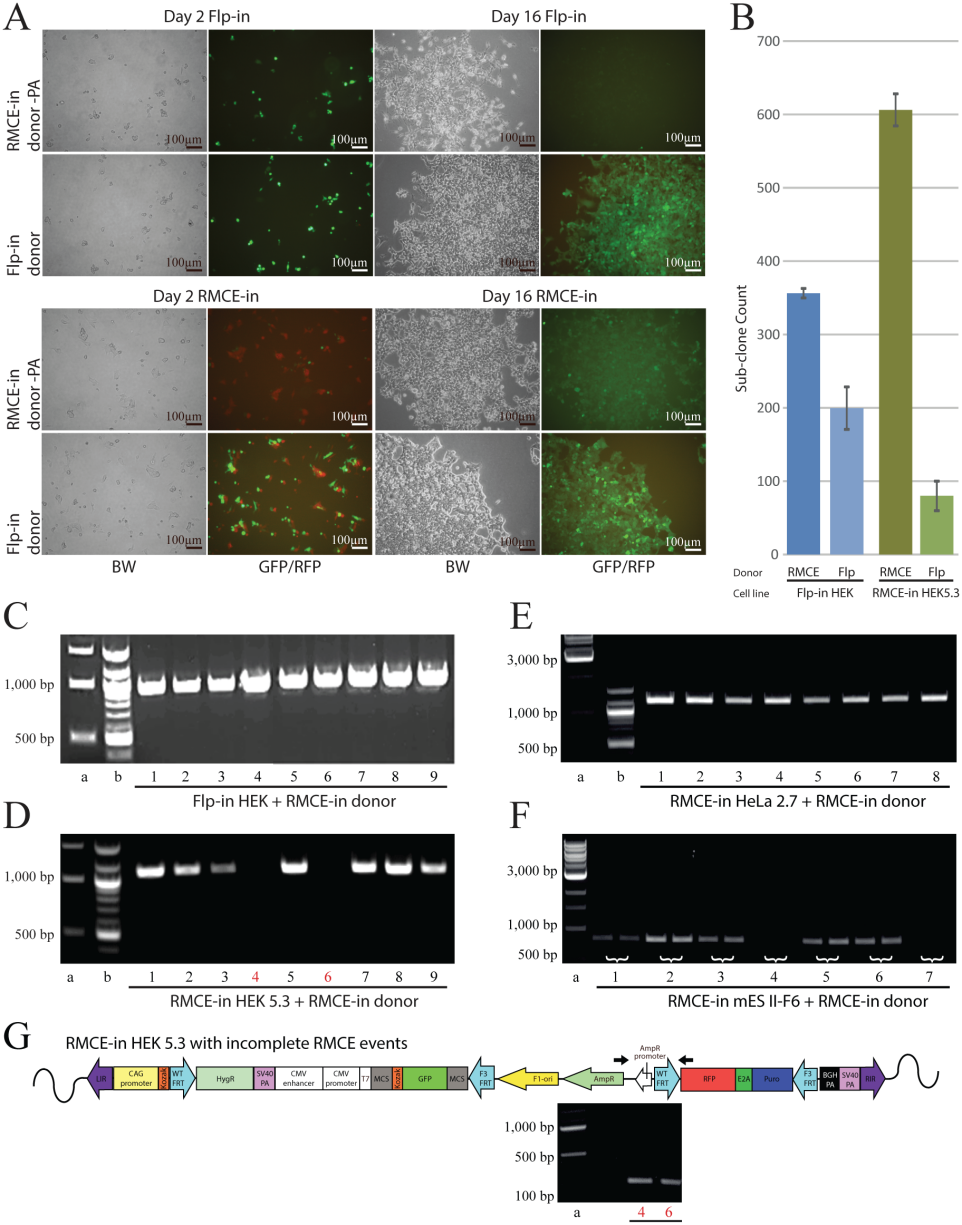
Comparison of RMCE-in HEK and Flp-in HEK cells. **(A)** Fluorescent and bright field pictures (BW) of RMCE-in HEK cells and commercial Flp-in^™^293 cells transfected with the RMCE-in donor (top row) or the Flp-in donor (bottom row) acquired at day 2 (transient) and day 16 (stable) post-transfection. Emission acquired under 1s exposure and 100X magnification with GFP and RFP overlay. RFP^+^ RMCE-in HEK cells with transient GFP established a GFP^+^/RFP^-^ colony at day 16 (HygR selection). With the RMCE-in HEK cells, the Flp-in donor emits more GFP than the RMCE-in donor. No GFP is observed when the RMCE-in donor is used in the commercial Flp-in HEK cell line. (**B)** Comparison of RMCE-in and Flp-in systems ability to form colonies using either RMCE or Flp donor. Colony count of three individual experiment with ± SD. (**C-F)** PCR-validated gene shift in Flp-in (C), RMCE-in (D) HEK5.3, HeLa2.7, and mES II-F6 (duplex load) cells using the RMCE-in donor. Flp-in and HeLa2.7 show gene-shift in all sub-clones, HEK5.3 and mES II-F6 show 7/9 and 5/7 sub-clones with gene shift, as verified with Sanger sequencing. RMCE-in HEK5.3 and mES II-F6 sub-clone-absent PCR product indicates an incorrect gene shift. (**G)** *Top*: Schematic representation of incomplete RMCE. Integration into the FRT, site but not subsequent excision at the two F3 sites results in a scenario similar to that seen in Flp-in. CAG expresses HygR, but no RFP, generating resistance-RFP-negative clones. The false negative sub-clones are revealed by amplifying the region from AmpR to RFP in the RMCE-in docking site (represented by black arrows). *Bottom*: Positive PCR product from sub-clone four and six (HEK5.3 with the RMCE-in donor) is indeed, an example of such a scenario.

In order to test the versatility of the new RMCE-in HEK293 cells, we compared the HEK clone 5.3 to the Flp-in^™^-293 cell line. For this purpose, we produced two donor plasmids containing a green fluorescent protein (GFP) gene as an example of a GEI. The first plasmid, named Flp-in donor, is identical to the pcDNA/FRT except for the integrated GFP (Fig 1B left). The second donor plasmid, named RMCE-in donor, was a modified version of the pcDNA/FRT in which the FRT site and the hygromycin-resistant gene (HygR) were moved upstream of the CMV promoter and the bGH polyadenylation (poly(A))-signal was removed. In addition, a multiple cloning site and a mutated FRT site, called F3, were placed downstream of GFP in the RMCE-in donor (Fig 1B right). In contrast to the Flp-in donor, the GFP in the RMCE-in donor does not contain a poly(A)-signal. Therefore, expression of GFP should be dependent on the poly(A)-signal from the docking site in the RMCE-in cell line. The RMCE-in and the Flp-in cell line were transfected with RMCE and Flp donors and a transient GFP expressions was observed along with a transient RFP from the RMCE docking site (Fig 6A day 2). The GFP expression from the RMCE-in donor was dim and weaker than the expression from the Flp-in donor, probably due to the lack of a poly(A)-signal (Fig 6A). Colonies formed with both cell lines at day 16 of HygR selection, with clear GFP expression from the Flp-in donor. A faint GFP signal from the RMCE-in donor in RMCE-in cells was observed, while the Flp-in cells expressed no GFP. In addition, the red fluorescence was lost in the RMCE-in colonies indicating a genuine gene shift (Fig 6A). As the genuine donor integration result in HygR expression, the colony forming efficiency of the RMCE-in and the Flp-in cell line transfected by either of the RMCE and Flp donors was evaluated at day 16 of HygR selection (Fig 6B). The RMCE-in donor performed better than the Flp-in donor in both the Flp-in and RMCE-in cell lines. However, the RMCE-in donor was distinctly more efficiently in the HEK 5.3 RMCE-in than the Flp-in cells (Fig 6B). This is most likely duo to the virtually irreversible recombination driven by the excess of donor and the different recognition sites, F3 and WT FRT, explaining the increased efficiency [9], as illustrated in Fig 1 by vertical equilibrium arrows. Also, the presence of the plasmid backbone when using the Flp-in donor might affect expression of the HygR.

Nine sub-clones were analyzed by PCR to confirm a genuine gene shift obtained with the RMCE-in donor. The forward primer was placed in the GFP gene and the reverse primer in the flanking genomic region of the RMCE-in cell line outside RIR (black arrows Fig 1C right). Since the genomic position of the Flp-in docking site is unavailable in the Flp-in^™^-293 cell line, we used a forward primer in the SV40 promoter and a reverse primer in HygR (black arrows Fig 1C left) in order to reveal if the RMCE-in donor was flipped in. Positive PCR products were observed from all nine sub-clones produced with the Flp-in cell line (Fig 6C). Using the RMCE-in cell line, we showed a band of the expected size (Fig 6D) in all colonies except sub-clone 4 and 6. Sequencing confirmed correct gene shift events. To investigate sub-clone 4 and 6 a PCR amplifying the sequence between the AmpR and the RFP gene was performed. Sequencing of the PCR product revealed Flp-in-like integration in the two sub-clones, however stalled at the subsequent excision at the two F3 sites required for complete RMCE (Fig 6G).

**Fig 7:**
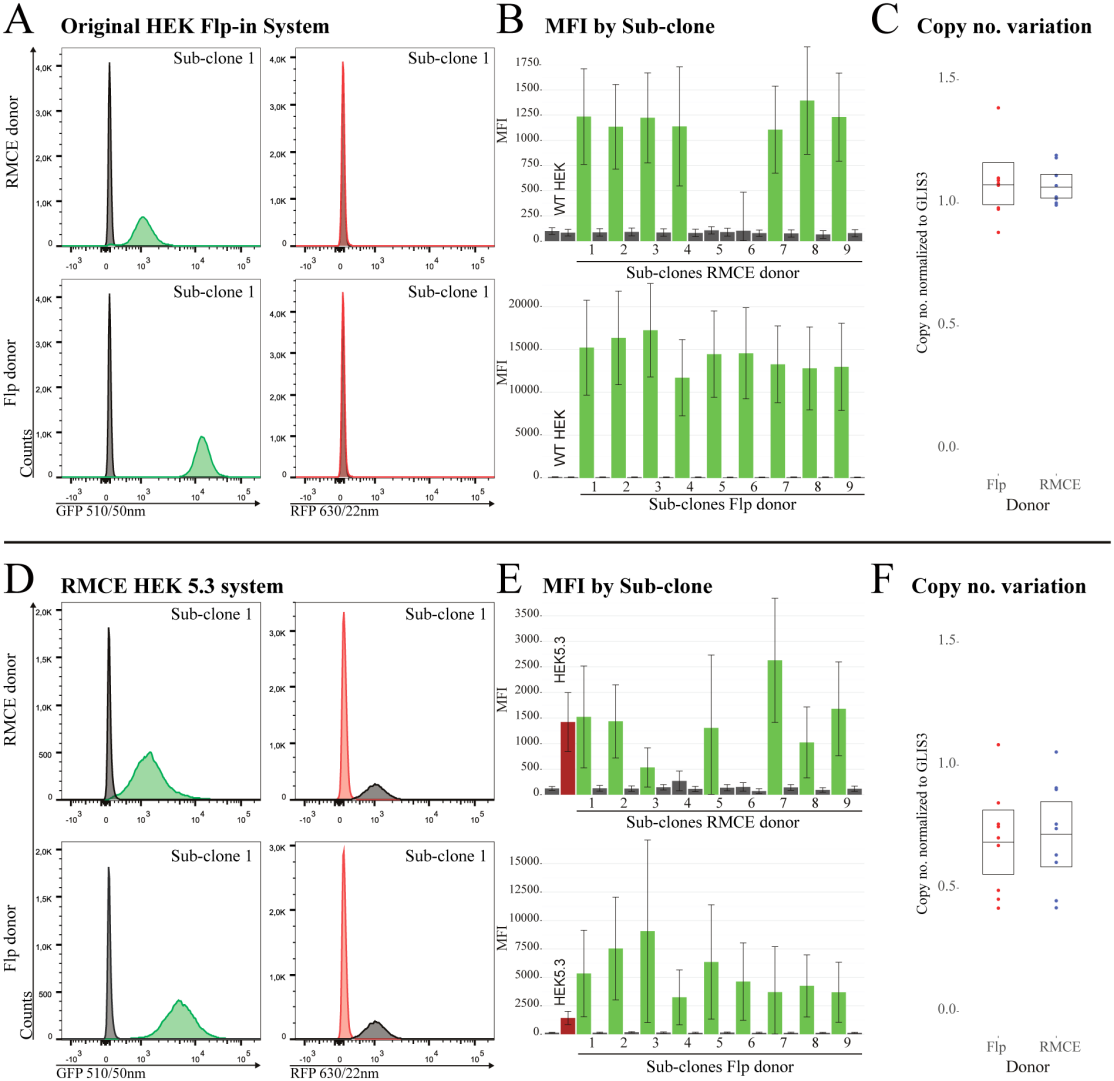
Expression in Flp-in^™^ and RMCE-in HEK after gene-shift. **(A)** Representative flow cytometry results of Flp-in^™^ sub-clone 1 using the HEK Flp-in system testing the RMCE (upper) and Flp (lower) donor compared with WT HEK (grey). **(B)** The RMCE (upper) and Flp (lower) sub-clones RFP and GFP MFI with SD. Cell singlet mean [min-max] counts; Flp sub-clones 20988[16977–23555], RMCE sub-clones 20002[16865–23680]. **(C)** The copy number normalized to GLIS6 of either Flp (red) or RMCE (blue) gene-shifted sub-clones. Non-parametric bootstrap for confidence limits (upper and lower bar) for the populations mean (middle bar). **(D)** Representative flow cytometry results of RMCE-in HEK sub-clone 1 using the HEK RMCE-in system testing the RMCE (upper) and Flp (lower) donor compared with HEK5.3 (grey). **(E)** The RMCE (upper) and Flp (lower) sub-clones RFP and GFP MFI with SD. Cell singlet mean [min-max] counts; Flp sub-clones 15312[104–19093], RMCE sub-clones 20168[16867–24292]. **(F)** The copy number normalized to GLIS6 of either Flp (red) or RMCE (blue) gene-shifted sub-clones. Non-parametric bootstrap for confidence limits (upper and lower bar) for the populations mean (middle bar)

The RMCE-in HeLa and mES cell clones were transfected with the RMCE-in donor plasmid and subsequently selected with HygR. To examine if the donor plasmid had been targeted to the RMCE-in docking site in the HeLa and murine clones, eight HeLa and seven murine HygR-resistant sub-clones were analyzed by PCR. All of the HeLa sub-clones and five of the seven mES cell sub-clones showed correct targeting (Fig 6E and 6F). The RFP and GFP expression levels in the gene shifted Flp-in and RMCE-in HEK sub-clones were analyzed by flow cytometry Flp-in (Fig 7A) or RMCE-in (Fig 7D). In The RMCE-in system, no RFP signal was detected in the RMCE sub-clones, as expected. Sub-colones 4 and 6 lost their RFP emission which indicates that the RMCE-in donor plasmids were, indeed, integrated in the RMCE-in docking site (Fig 7E). MFI values for GFP fluorescence were recorded for nine sub-clones created using both donors in both cell lines (Fig 7). GFP expression could also be observed in seven out of nine sub-clones produced using the RMCE-in donor in the Flp-in cell line (Fig 7A). Using the RMCE-in donor in our RMCE-in cell line gave a clear signal in all sub-clones except 4 and 6 (Fig 7A), which corresponds to the observation from the PCR (Fig 6D+6G). The GFP gene is not expressed as the poly(A)-signal in the RMCE-in docking site cannot be trapped in such a scenario. Using the Flp-in donor, where GFP has its own poly(A)-signal, generated higher MFI than using the RMCE-in donor for all nine analyzed sub-clones in both cell lines (Fig 7). To test if this was due to additional random integrations, we analyzed the copy number for each set of nine sub-clones using qPCR (Fig 7C+7F). A randompattern of various copy numbers between the sub-clones was observed for all sets independently of the donor plasmid and the cell line. This indicates that besides the target integration into the docking site, additional integrations are, indeed, present after gene shift in both the HEK Flp-in and the HEK RMCE-in cell line. Unpaired 2-tailed Students t-test depicts no significant difference using either of the systems or donors (Flp-in p = 0.76 and RMCE-in p = 0.75). However, the lower GFP expression from the RMCE-in donor indicates that the lack of a poly(A)-signal can, indeed, reduce the contribution of random integrations to the transgene expression.

Similar pattern are seen in each diagram independently of cell line and donor plasmid combination. (**A)** Flp-in^™^-293 cell sub-clones made by transfection with either the RMCE-in or the Flp-in donor plasmid. Sub-clone 5 and 6 of the RMCE donor was GFP-negative. (**B)** RMCE-in HEK5.3 cell sub-clones made by transfection with either the RMCE-in or the Flp-in donor plasmid. The basal RFP emission from the HEK5.3 presented as gray histogram (MFI 1141) shows evidence of loss of RFP upon gene shift in RMCE-in cells. Sub-clone 4 and 6 of the RMCE donor were GFP- and RFP-negative as suspected from the incomplete RMCE (Fig 6D and 6G). We generally observed a higher GFP MFI of the RMCE donor in RMCE-in than the Flp-in^™^-293.

## Discussion

Here we describe three different RMCE-in cell lines and a protocol for the generation of new cell lines as an alternative to the Flp-in cell line systems. The loss of RFP upon targeted GEI integration, which is possible in our RMCE system was used to indicate a successful exchange of the transgene. A RFP reporter in the parental cells is advantageous to evaluate transgene expression at a given insertion locus and to uncover whether the clones are homogenous or chimeric. The fluctuating RFP expression levels post transposition confirms the well-known positional effect flanking genomic sequences inflict on transgene expression. The phenomenon was observed in HeLa clones with a single integration showing a several-fold difference in RFP expression e.g. comparison between HeLa 2.7 and HeLa 3.6 (Fig 3). Moreover, HEK 1.6 harbored four integrations with twice the RFP expression than HEK 5.3 which contained a single integration. This non-linear relation between copy number and expression was also found in mES cells presenting equal MFI between clones of single and double integrations. Using transposition to establish a RMCE-in docking site avoids integration of a plasmid backbone, and ease downstream verification of potential clones, constituting new RMCE-in cell lines. LDI mapping of transposon integration utilize the known gene-cassette termini and alleviate the choice of primers and restriction enzymes. The RMCE-mES cells however, was created by electroporation increasing the possibility of single-integration and therefore not avoiding integration of plasmid backbone. Linearization was performed outside LIR/RIR since exonucleases have been shown to gradually shorten the constructs. In the presented study, we used the original SB10 transposase rather than a hyperactive transposase such as SB100X. SB10 is preferable to establish single integration using fairly small (4.3 KB) transposon. However it may be more beneficial to use SB100X in cell lines that are difficult to transfect or if the transposition rate is too low.

Even though both the Flp-in and the RMCE-in donor plasmid can be used in the RMCE-in cells, the RMCE-in donor was more efficient. This is most likely due to the virtually irreversible recombination inherited in the RMCE-in system, but possibly also a positive effect of avoiding bacterial backbone. We consider the fact that, if wanted, a first RMCE step may be followed by any other RMCE-based modification as one of the major advantages explaining the exceptional role of Flp-RMCE. The advantages of this system over Ser-recombinases or even other Thr-recombinases [9]. It may motivate further applications as introduced in the present paper. The gene shift copy number analysis of Flp and RMCE donor revealed that both Flp and RMCE donors integrated more than one copy at times. However the effect from additional integration is negligible in the RMCE-in system due to both promoter and polyA trapping. RMCE-in HEK 5.3 sub-clone 4 and 6 did not show a genuine gene shift using the RMCE-in donor (Fig 6D) and may be explained by limited FlpO activity at subsequent excision between the two F3 FRT sites. Faulty RMCE events can easily be identified by PCR, and may be prevented optimizing the Flp to donor ratio in the transfection.

The RMCE-in system includes poly(A)-trapping opposite the donor verified by loss of RFP and gain of GFP expression in targeted sub-clones in the RMCE-in cell lines. We did however observe a minor GFP expression when using the RMCE-in donor in the Flp-in cell line (Fig 7A day 16). This could be due to an upstream cryptic poly(A) sequence since GFP contains no poly(A) in the RMCE-in donor [15] explaining why a minor and transient GFP expression was observed from the RMCE-in donor plasmid (Fig 6A day 2). RMCE-in HEK5.3 sub-clones 4 and 6 with partial recombination show no GFP due to missing polyA (Figs 6G and 7E). A previous study has shown that mRNA can be translated, without polyadenylation, but is degraded rapidly [16]. To disentangle the transient GFP signal the RMCE-in donor plasmid was sequenced and revealed an AATAAA sequence 1080 bp downstream f1-origin and into the end of AmpR-resistant gene in the plasmid backbone, which may serve as a poly(A)-signal.

A similar RMCE-in system was earlier successfully introduced *in vivo* in a transgenic pig model [17]; and several other RMCE systems have been developed in mouse models carrying the RMCE-in donor site [18–20]. This system however eases the use of previously used GEI Flp donors and simplifies validation of knock-in effects before a labor-intensive targeted approach. The RMCE-in system is comparable or even more efficient in integrating donor constructs. Nonetheless, we see that main advantage of the system in the fact that it is a non-proprietary contribution to the scientific community that is freely distributed. The RMCE-in construct and cell lines are now freely available without restrictions from Addgene and the ATCC cell bank for the benefit of scientific community.

## Materials and Methods

### Vector construction

The RMCE-in SB transposon plasmid (addgene #67275) was constructed from the floxed-Ei-Ubi-*PSEN1M146I* plasmid described in Jakobsen et al. [17]. The CAG and Ubi promoters were exchanged by *SnabI* and *PacI* digested CAG-GFP and floxed-Ei-Ubi-*PSEN1M146I* plasmids. The F3, multiple cloning site, and poly(A)-signal (Synthesized from Genscript) was inserted into the floxed-CAG-*PSEN1M146I* plasmid using *ClaI* and *SbfI*. The *PacI*-FRT*-*Kozak-RFP-E2A-Puro-F3-*ClaI* (Synthesized from Genscript) was inserted into the floxed-CAG-*PSEN1M146I*-F3-PA plasmid using the *PacI and ClaI* to produce the floxed-CAG-FRT*-*Kozak-RFP-E2A-Puro-F3-PA and loxP sites were removed by *SpeI-EcoRV*. The RFP refers to the far-red fluorescent protein *Katushka* [21].

The RMCE-in donor plasmid (addgene #67276) was constructed as follows: A fragment consisting of a Kozak element, a GFP variant *mWasabi* [22], a multiple cloning site (MCS), and a F3 reverse FRT^**(G)**^ was inserted into the pcDNA5/FRT vector using *XhoI* and *ApaI*. The FRT sequence and the HygR was cut out of pcDNA5/FRT using NgoMIV. FRT and HygR was then PCR-amplified and reinserted upstream of the CMV promoter in pcDNA5/FRT vector using *MfeI* and *NruI*. The bGH poly(A)-tail was cut out using *PciI*. The Flp-in donor plasmid was constructed by cutting out the GFP gene from the RMCE-in donor plasmid using *NheI* and *BsrGI*, and then inserting GFP into the pcDNA5/FRT vector using *NheI* and *Acc65I*.

### Transfection of cells for insertion of the RMCE-in docking site

HEK293 (ATCC-CRL-1573) and HeLa (ATCC-CCL-2) cells were acquired from the ATCC cell bank. The cells were seeded in a 12-well plate and grown in DMEM media with 10% fetal bovine serum and 1% penicillin-streptomycin at ~50% confluence. Cells were co-transfected with the RMCE-in transposon and SB10 Sleeping Beauty transposase using X-tremeGENE 9 DNA (Roche). Amount and ratio of the transposon and transposase plasmid was added in three different quantities as seen in Table 1.

**Table 1:** 

Plasmid/well no.	1	2	3	4	5	6
RMCE-in transposon	500 ng	125 ng	30 ng	500 ng	125 ng	30 ng
SB10 transposase	50 ng	12.5 ng	3 ng	25 ng	6.25 ng	1.5 ng

Cells were split in 80/20% transferred to separate 60 cm^2^ petri dishes the second day. The puromycin selection (1 μg/ml) was initiated day 3 and renewed every 3-4^th^ day. HEK colonies were selected for 18 days and HeLa colonies for 11days before harvested. The mES cell colonies were made from 20 μg *PvuII* linearizing RMCE-in transposon, electroporated into 10 million TM219cells [23]. ES cells were seeded on mitotically inactivated murine fibroblasts and selected for 5 days with puromycin (0.6 μg/ml).

### Southern blot

Southern blot was performed as previously described Jakobsen et al. [24]. DNA was digested overnight at 37°C; with *EcoRV* and *KpnI* or *PstI*. To increase fluidity DNA was heated 30 min at 56°C before gel electrophoresis. The 700 bp dCTP^32^ RFP probe, was isolated from the RMCE-in transposon plasmid using *KpnI* and *BsrGI*.

### Flow cytometry

Flow cytometry was performed on selected clones from HEK, HeLa and, mES cells. The cells were washed twice in PBS before Trypsin-EDTA treatment. Detached cells were centrifuged at 320g, and the pellets were suspended in 500μl PBS + 0.1% BSA. The clones were analyzed on a LSRFortessa (BD) flow cytometer using the 488nm and 561nm laser with 510/50nm and 630/22nm band pass, respectively. All data analyses were performed with the FlowJo software (v.X.0.7, Tree Star Inc., Ashland, OR, USA). Cell singlet population analysis was performed by gating voltage area/height ratio, on both the forward scatter and side scatter to omit aggregating cells. The median fluorescent intensity was calculated on the population of singlets with ± SD values calculated on both RFP and GFP histograms.

### Long-distance inverse (LDI)-PCR

LDI-PCR was performed as previously described in Jakobsen et al. [24]. Briefly, 0.5 μg of purified DNA isolated from clones was digested with *BsrGI* and *Acc65I*, creating compatible cohesive ends, at 37°C for 3 h and heat inactivated at 80°C. Ligation by T4 ligase, was performed overnight at 14°C. PCR primers: Fwd: 5’-CAGCCATTGCCTTTTATGGT-3’, Rev: 5’-AAATACAAAATTGGGGGTGG-3’ and nested primer Fwd: 5’-GCTGGTTGTTGTGCTGTCTC-3’, Rev: 5’-GGGCGTACTTGGCATATGAT-3’.

### Quantitative PCR on genomic DNA

The absolute copy number of GFP and hygromycin was determined using qPCR. The reaction was run in a clear 96-well plate using 50ng genomic DNA (25 ng/μl), SYBR GREEN and 3 *p*mol forward and reverse primer in a total volume of 10 μl and analyzed on a Light cycler ^®^ 480 (Roche). Cycle conditions were: 95°C, 10 s; 63°C, 20 s; 72°C, 30 s; 40 repeats. The GFP cycle number was correlated to endogenous *GLIS3* in the triploid HEK and HeLa cells. The qPCR primers *GLIS3* (5’-CCACACTACCCCGATTCC-3’ and 5’-TGTAATGCCCGAGTGAGTCG-3’), GFP (5’-TCAACCTGGAGGTGAAGGAG-3’ and 5’-TACTTGGTGAAGGCCCTGTT-3’) and HygR (5’- -3’ and 5’- -3’)

### Transfection of the RMCE-in and Flp-in^™^-293 cell lines

RMCE-in HEK293 or HeLa and Flp-in^™^-293 (Life Technologies #R750-07) were seeded at 60% confluence in a 12-well plate and all transfections were made in triplicates. Cells were transfected with 250 ng donor and PGK-FlpO plasmid (5067 bp) or 250 ng empty pUC 19 control plasmid using X-tremeGENE 9 in HEK293 or XtremeGENE HP (Roche) in HeLa cells. All cells were transferred using 0.05% Trypsin-EDTA (Gibco) to 60 cm^2^ petri dishes the following day. Day three, cells were subject to HygR selection (100 μg/ml for HEK293 cells and 250 μg /ml for HeLa cells) and continuously HygR renewed every 3-4^th^ day until harvested. Six million murine RMCE-in ES cells were electroporated using 10 ug donor and 10 ug FlpO plasmid (5067 bp) or 10 ug empty pUC 19 control plasmid. ES cells were seeded on mitotically inactivated murine fibroblasts and selected for 3 days with HygR (125 μg/ml).
